# Exploring the paradox: double burden of malnutrition in rural South Africa

**DOI:** 10.3402/gha.v6i0.19249

**Published:** 2013-01-24

**Authors:** Elizabeth W. Kimani-Murage

**Affiliations:** 1 African Population and Health Research Center, Nairobi, Kenya; 2 School of Public Health, Faculty of Health Sciences, University of the Witwatersrand, Johannesburg, South Africa

**Keywords:** nutrition transition, double burden of malnutrition, stunting, underweight, wasting, overweight, obesity, metabolic disease risk, HIV, low- and middle-income countries, South Africa

## Abstract

**Background:**

This article is a review of the PhD thesis by Elizabeth Kimani-Murage that explores the double burden of malnutrition in rural South Africa. This is in the context of a worryingly rapid increase in obesity and obesity-related diseases in low- and middle-income countries (LMICs) including South Africa, and in the wake of on-going nutrition transition and lifestyle changes in these countries.

**Objective:**

To understand the profiles of malnutrition among children and adolescents in a poor, high HIV prevalent, transitional society in a middle-income country.

**Methods:**

A cross-sectional growth survey was conducted in 2007 targeting 4,000 children and adolescents aged 1–20 years. In addition, HIV testing was carried out on children aged 1–5 years and Tanner pubertal assessment among adolescents aged 9–20 years.

**Results:**

The study shows stunting at an early age and adolescent obesity, particularly among girls, that co-exists in the same socio-geographic population. The study also shows that HIV is an independent modifiable risk factor for poor nutritional outcomes in children and makes a significant contribution to nutritional outcomes at the individual level. Significant predictors of undernutrition at an early age, documented at individual, household, and community levels, include child's HIV status, age and birth weight, maternal age, age of household head, and area of residence. Significant predictors of overweight/obesity and risk for metabolic disease during adolescence, documented at individual and household levels include child's age, sex, and pubertal development, household-level food security, socio-economic status, and household head's highest education level.

**Conclusions:**

The combination of early stunting and adolescent obesity raises critical concerns in the wake of the rising public health importance of metabolic diseases in LMICs. This is because, both paediatric obesity and adult short stature are risk factors for metabolic syndrome and metabolic diseases in adulthood. Clearly, policies and interventions to address malnutrition in this and other transitional societies need to be double-pronged and gender-sensitive.

Nutrition transition, being experienced in low- and middle-income countries (LMICs) undergoing rapid economic transition and urbanisation, is a major driving force behind the increase in levels of obesity in LMICs, despite persistence of undernutrition (1, 2). Therefore, both undernutrition- and obesity-related diseases contribute substantially to the burden of disease in these societies (3). The problem of obesity is not only experienced among adults but also in children (4). Childhood obesity is the driving force behind paediatric metabolic syndrome risk that has become a growing public health concern in LMICs (5). Childhood obesity is associated with short-term health problems including heightened risk of psychosocial morbidity, cardiovascular complications and type 1 and type 2 diabetes. It is also associated with long-term problems including obesity and cardio-metabolic diseases and impaired social and economic productivity in adulthood (6).

Due to its historical background, characterised by nearly half a century of Apartheid, high levels of HIV/AIDS over the past few decades (7), and the recent rapid economic and social transition and urbanisation (8, 9), South Africa has undergone a complex health transition (10–13). It is characterised by high levels of persisting undernutrition among the Black population (13), potentially due to high levels of food insecurity reported at the household level (14). On the other hand, a rapid nutrition transition has been experienced in the country with a marked shift from staple foods towards an energy dense diet occurring alongside urbanisation (15, 16). High levels of physical inactivity and sedentary lifestyles have also been associated with the nutrition transition in several studies in South Africa (16, 17). This has resulted in a high prevalence of overweight and obesity among adults, particularly women; for example, 55% of adult women are either overweight or obese, with a consequent high disease burden of non-communicable diseases (10–13).

Evidence of obesity among children and adolescents is emerging though still limited, and little is known about co-existence of undernutrition with obesity among children in the same geographical setting (18, 19). This co-existence is the focus of this study. This article presents a review of a PhD thesis based on a study whose main objective was to better understand the profiles of malnutrition among children and adolescents in a poor, high HIV prevalent transitional society in a middle-income country and in so doing, to inform policies and interventions. The results are organised into three thematic areas including: (1) patterns of malnutrition; (2) adolescent obesity and risk for metabolic disease; and (3) child undernutrition in the context of HIV. The different themes are distilled from four studies emanating from the doctoral research (20–23).

## Conceptual framework

**Fig. 1:**
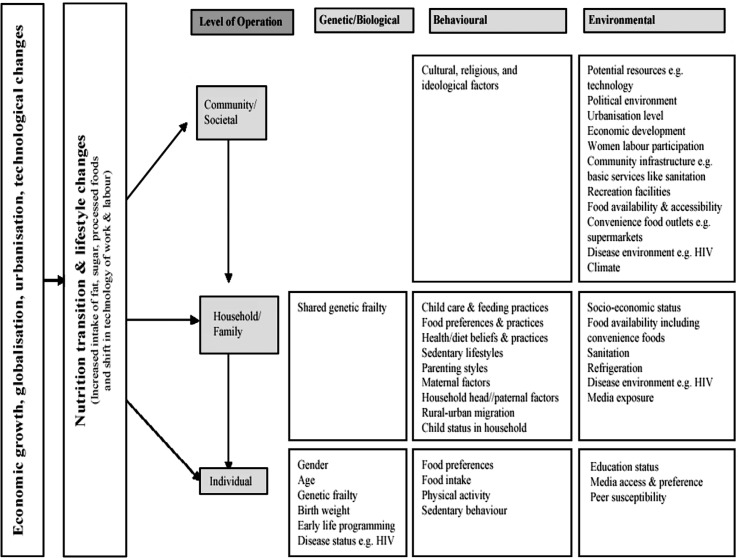
Conceptual framework on nutrition transition and hierarchical organisation of factors influencing a child's nutritional status. Note: The framework is adapted from Popkin's (2003) model of nutrition transition (2), and Griffiths (2004) framework of interpreting community, family and individual effects on child weight status (24). It is also informed by the Food and Agriculture Organization's (FAO)'s (2004) model on changes in food systems (25), and Davison's (2001) ecological model for childhood obesity (26).

Figure 1 presents a conceptual framework illustrating the hierarchical organisation of the different societal levels that influence a child's nutritional status (2, 24–26). The framework recognises that nutrition transition and lifestyle changes, occurring in transitional societies, influence a child's nutritional status at different societal levels. Nutrition transition and lifestyle changes influence nutritional status directly at individual level. They also have an indirect influence through changes experienced at household and community levels. In the reviewed thesis, children's nutritional status in a transitional society is described. Furthermore, distal factors influencing a child's nutritional status are assessed at the individual, household, and community levels.

## Methods

### Study setting and population

This study was conducted in the Agincourt sub-district, Mpumalanga Province, rural northeast South Africa, alongside South Africa's border with Mozambique. Agincourt is a semi-arid setting, situated in the former Gazankulu homeland. The study was nested within the Agincourt health and socio-demographic surveillance system (HDSS) of the University of the Witwatersrand. The Agincourt HDSS is a multiround prospective community study, which was established in 1992, and covers the entire Agincourt sub-district. Until 2007, when the site was extended, the HDSS followed some 70,000 people living in 11,500 households in 21 villages where about 30% were of Mozambican origin. The area is characterised by high levels of unemployment and poverty (27). Labour migration, mainly circular rural–urban migration, is widespread involving up to 60% of working age men and growing numbers of women (28). Being in a former homeland, the land is subdivided into plots too small to support subsistence farming. Piped water is available at community level, but there are frequent water shortages in most villages. Sanitation is poor, particularly in the former refugee settlements (29). Literacy levels have improved post-apartheid in the younger generation, but high illiteracy levels remain among the older generation, reaching levels of almost 80% for those aged 60 years and above (30). Health care services are limited. The area is characterised by a high prevalence of HIV/AIDS – a third of pregnant women visiting public antenatal health clinics in the province are infected (7). The study area, Agincourt HDSS, and local demographics are described in detail elsewhere (29).

### Data sources

The study used data from three sources: growth survey in 2007, a follow-up study in 2008, and the Agincourt HDSS.

#### Growth survey (2007)

A growth survey nested within the Agincourt HDSS was conducted between April and July 2007 (20, 22, 23). The study sample comprised children and adolescents aged 1–20 years, selected from the entire population, and 4,000 children and adolescents were targeted, comprising 100 males and 100 females for each year of age. We oversampled 10–15 children per age–sex group to counter possible non-participation. Thus, a total of 4,658 children were randomly selected. Only children who had lived in the study area at least 80% of the time since birth, or since 1992 when enrolment in the Agincourt HDSS began were included. The study involved anthropometric measurements (height, weight, and waist circumference) according to standard procedures (31). Additionally, pubertal assessment of adolescents aged 9–20 years using the Tanner 5-point pubertal self-rating scale (32), and HIV testing of children 1–5 years using two concurrent rapid tests: Uni-Gold™ (Trinity Biotech, Bray, Ireland) and Determine™ (Abbott, Wiesbaden, Germany) in accordance with WHO recommendations for HIV screening in children (33) were carried out.

#### Follow-up study (2008)

A follow-up of a subset of the 2007 growth survey was conducted between May and June 2008 (21). This involved follow-up of HIV-infected children aged 1–5 years identified in the 2007 survey (*n*=35). In-depth interviews with caregivers of HIV-infected children who were aware of the child's status (*n*=22) were conducted. The in-depth interviews explored issues on attitudes, reactions and the impact of knowing a child's HIV status, caregiving and seeking antiretroviral treatment (ART) for the child, and challenges in caregiving.

#### Agincourt HDSS

The Agincourt HDSS is a longitudinal community surveillance that involves systematic annual recording of vital demographic events, including births, deaths, and in- and out-migrations occurring in the entire Agincourt sub-district. Additional data to provide information on particular areas of interest, for example, food security is collected as special census modules nested within the annual update rounds. An asset survey conducted in each household every 2 years gives a measure of household socio-economic status (SES) (29). Data on potential explanatory variables were extracted from the Agincourt HDSS including: individual factors: child's age, sex, birth weight, and relationship to household head; household factors: mother's age, nationality, highest education level, marital/union status, co-residence with child and place of delivery (for index child aged less than 5 years), and household head's age, sex and highest education level, household food security and SES; and area of residence as a proxy for community-level factors.

### Data analysis

#### Outcome measures

This included height-for-age z scores (HAZ), weight-for-age z scores (WAZ), weight-for-height z scores (WHZ), stunting, underweight, wasting, body mass index (BMI), overweight, obesity, waist-to-height ratio, and central obesity. HAZ, WAZ, and WHZ for children up to 60 completed months were generated using the WHO 2006 growth standards while for those aged 5–17 years were determined using the NCHS/WHO reference. Stunting, underweight, and wasting were, respectively, defined as z-scores less than −2 (34). BMI was determined by dividing weight (in kg) by height squared (in metres). Overweight and obesity in children aged 2–17 years was determined using the absolute age and sex-specific cut-offs for BMI recommended by the International Obesity Task Force (IOTF) (35). For adolescents aged 18–20 years, adult cut-off points of BMI ≥25 and ≥30 kg/m^2^ for overweight and obesity were used (36). Dividing waist circumference by height generated waist-to-height ratio. Waist-to-height ratio cut-offs of 0.5 for both sexes (37) were used to determine those with central obesity, hence risk of metabolic disease among adolescents.

#### Explanatory variables

We included individual-level characteristics (age, sex, birth weight, HIV status [for children aged 1–4 years, although HIV test was done for children 1–5 years], pubertal stage [for adolescents], and relationship to household head); household-level characteristics including maternal characteristics (age, nationality, highest education level, marital status, co-residence with child, and place of delivery), and other household characteristics (household head's age, sex, and highest education level, household food security and SES); and community-level characteristics (area of residence).

#### Descriptive analysis

We used described patterns of malnutrition for 1–20 year olds by age and sex; by HIV status for children aged 1–4 years; and by pubertal stage for adolescents aged 9–20 years (20, 22).

#### Multiple linear and logistic regression analysis

This was carried out with the outcome and explanatory variables described above to determine predictors of undernutrition in children aged 1–4 years, and predictors of overweight/obesity and risk for metabolic disease for adolescents aged 10–20 years. Only variables significantly associated with nutritional status from the univariate analyses at the 10% level of significance were included in multiple regression analysis. Significant association was determined at the 5% level of significance (95% confidence interval) (22, 23).

#### Qualitative data analysis

This was conducted across all transcripts using a constant comparative method, to identify themes and their repetitions and variations (21, 38).

## Results

### Patterns of malnutrition

Analyses involved a total of 3,489 children: 1,724 (49.4%) males and 1,765 (50.6%) females, aged between 1 and 20 years (20).

**Fig. 2:**
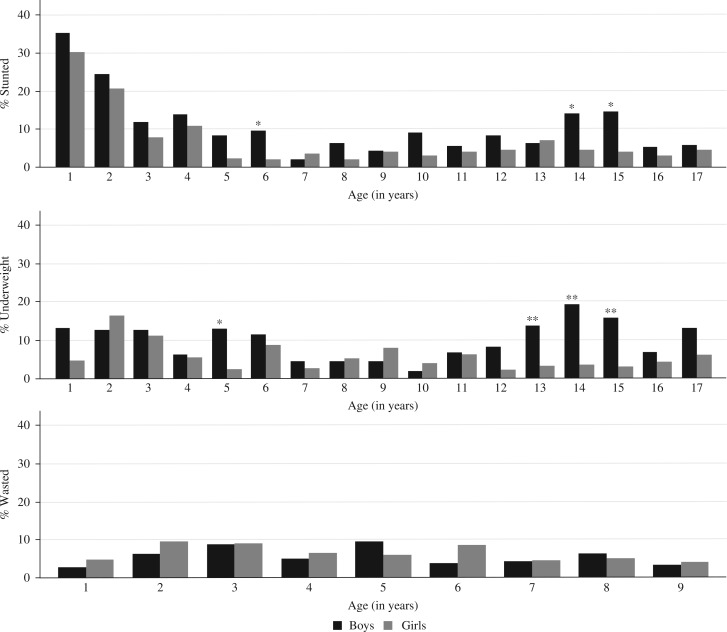
Prevalence of stunting and underweight for children aged 1–17 years (*n*=3,070) and wasting for children aged 1–9 years (*n*=1,641) by sex, Agincourt sub-district, South Africa, 2007. Significant difference by sex: *p-value < 0.05, **p-value < 0.01, ***p-value < 0.001. Source: Kimani-Murage et al. 2010 (20).

Stunting was prevalent, particularly in children aged less than 5 years (18%) and highest in children aged 1 year (32%). The prevalence was lowest among children aged 5–9 years (5%), but went up during adolescence particularly in boys between 14 and 15 years of age when the prevalence rose to 14–15%. The prevalence of being underweight was highest among children aged less than 5 years at 10%, and lowest among children aged 5–9 years (6%), and peaked in boys aged 14 years (19%). The prevalence of wasting was 7 and 6% in children aged 1–4 and 5–9 years, respectively. See Fig. 2 (20).

**Fig. 3:**
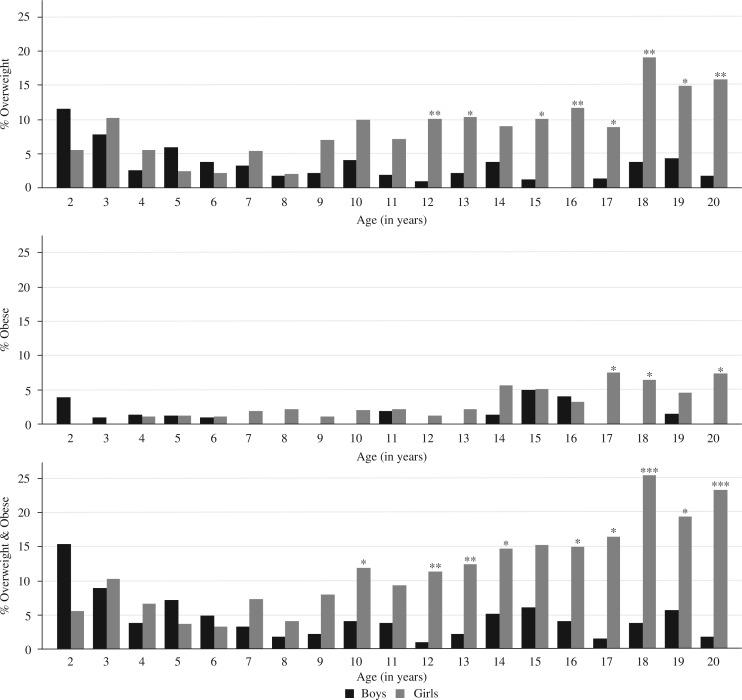
Prevalence of overweight, obesity & combined overweight and obesity for children aged 2–20 years (*n*=3,358) by age and sex, Agincourt sub-district, South Africa, 2007. Significant difference by sex: *p-value < 0.05, **p-value < 0.01, ***p-value <0.001. Source: Kimani-Murage et al. 2010 (20).

The prevalence of combined overweight and obesity was moderate in early childhood and low in late childhood, and remained so in older boys. The prevalence rose progressively in girls aged 10 years and older. The prevalence was highest amongst adolescents in the age category 15–20 years (12%) and averaged 19% in girls, compared to 4% in boys, reaching 25% at age 18 years in girls. The prevalence was significantly higher in girls than in boys in most of the adolescent years (*p*<0.05, respectively, see Fig. 3) (20). With regard to Tanner staging, combined overweight and obesity was lower in the earlier stages of puberty, but increased markedly during the later stages in girls (from 7% at stage 1 to 35% at stage 5); while in boys the prevalence remained low (<5%) throughout the stages. The prevalence of combined overweight and obesity was significantly different by sex at Tanner stages 3, 4 and 5 (*p*<0.05, respectively). About 10% of adolescents were potentially at risk of metabolic disease, which was significantly higher for girls (15%) than boys (3%) (*p*<0.001) (20).

### Adolescent obesity and risk for metabolic disease

The analysis for weight status and central obesity involved 1,848 participants aged 10–20 years: 903 (49.6%) boys and 945 (50.5%) girls. Age, sex and pubertal development status (individual level factors) all emerged as significant predictors of a child's weight status and central obesity, which are in turn risk factors for metabolic disease. Older children, girls, and pubertal and post-pubertal adolescents were more likely to be overweight/obese. At household level, among maternal factors included, the mother's age was a significant predictor of overnutrition, with children of mothers aged 50+ years more likely to be over nourished. Other significant predictors at household level included household head's highest education level, food security, and socio-economic status. Little education (less than secondary certificate) was associated with overnutrition. Additionally, food security and SES were both positively associated with overnutrition. Area of residence at community level was not significantly associated (see Table 1) (23).

**Table 1:** Predictors of weight status and central obesity among adolescents 10–20 years (*n*=1,848), Agincourt sub-district, rural South Africa, 2007

	BMI z-scores^2^	Overweight and obesity^3^	WHtR z-scores^2^	Central obesity^3^

Variable^1^	Coeff [95% CI]	OR [95% CI]	Coeff [95% CI]	OR [95% CI]
Child-level factors					
Child age	**−**0.0 [**−**0.0, 0.0]	1.0 [0.9, 1.1]	**−**0.2 [**−**0.0, 0.0]	1.1 [1.0, 1.2]*	
Child sex					
Boys (ref)	0	1	1	1	
Girls	0.6 [0.5, 0.7]***	4.2 [2.8, 6.4]***	−0.0 [−0.1, 0.1]	7.2 [3.7, 14.2]***	
Pubertal status					
Pre-pubertal	0	1	1		
Pubertal	0.3 [0.1, 0.5]***	1.4 [0.7, 2.8]	0.1 [**−**0.1, 0.2]	1	
Post-pubertal	1.1 [0.8, 1.4]***	4.4 [1.9, 10.1]***	0.4 [0.2, 0.7]***	2.9 [1.8, 4.6]***	
Household-level factors					
*Maternal factors*					
Mother's age category					
35–49 (ref)		1		1	
15–34		1.4 [0.8, 2.3]		1.9 [0.9, 4.0]	
50 +		1.5 [1.0, 2.2]		1.8 [1.1, 2.8]*	
*Other household factors*					
HHH education					
No education (ref)	0				
<Secondary level	−0.1 [−0.3, −0.0]*	0.6 [0.4, 0.9]*			
Secondary level and higher	0.1 [**−**0.1, 0.4]	1.1 [0.6, 2.0]			
Food security					
Not enough (ref)	0	1	1	1	
Enough	0.2 [**−**0.0, 0.3]	1.4 [0.8, 2.2]	0.1 [0.0, 0.2]*	1.7 [0.9, 3.2]	
Wealth index tertiles					
Lowest (ref)	0	1	0	1	
Medium	0.3 [0.1, 0.4]**	1.4 [0.9, 2.2]	0.0 [**−**0.3, 0.2]	1.3 [0.7, 2.2]	
Highest	0.3 [0.1, 0.4]***	2.0 [1.3, 3.1]**	0.1 [0.0, 0.3]*	1.9 [1.1, 3.3]*	

1 Only variables with significant association at 10% level of significance are shown.

2 Linear regression.

3 Logistic regression.

* *p*<0.05

** *p*<0.01

*** *p*<0.001.

### Child undernutrition in the context of HIV

This analysis involved 671 children aged 1–4 years in 2007: 338 (50.4%) boys and 333 (49.6%) girls.

#### Patterns of nutritional status by HIV status and predictors of undernutrition

Consent was given for HIV testing in 640 of the 671 children aged 1–4 years (95%). Six hundred and twelve were not infected with HIV, 28 were HIV infected, while 31 were not tested (no consent for the test) giving a prevalence of 4.4% (95% CI: 2.79–5.97). A negligible number of HIV-infected children were on ART. The mean HAZ, WAZ, and WHZ were all significantly lower in HIV-infected children compared to children not infected with HIV (*p*<0.05, respectively) (22).

In the multiple regression, HIV status was strongly associated with HAZ and WAZ, but its association with WHZ, stunting, underweight, and wasting was not significant at the 5% level. Other significant predictors of child's nutritional status included the child's age, birth weight, maternal age, age of household head, and area of residence. Generally, an increase in child's age was negatively associated with undernutrition, while low birth-weight (prevalent in about 10% of the children aged 1–4 years) was positively associated with undernutrition. Children of younger mothers and younger household heads were more likely to be under nourished. Additionally, children from villages predominantly inhabited by people of Mozambican origin were more likely to be under nourished (see Tables 2 and 3) (22).

**Table 2:** Linear regression analysis for predictors of height-for-age, weight-for-age, and weight-for-height z-scores among children aged 12–59 months (*n*=671), Agincourt, South Africa (2007)

	HAZ	WAZ	WHZ

Variable^1^	Coeff [95% CI]	Coeff [95% CI]	Coeff [95% CI]
Child-level factors			
HIV status			
Negative (ref)	0	0	0
Positive	−0.8 [−1.2, −0.3]**	−0.7 [−1.2, −0.3]**	**−**0.5 [**−**1.0, 0.1]
Unknown status	0.2 [**−**0.2, 0.6]	0.2 [**−**0.2, 0.6]	0.2 [**−**0.3, 0.7]
Child age	0.2 [0.1, 0.3]***		−0.2 [−0.3, −0.1]**
Birth weight			
≥ 2.5 kg (ref)	0	0	0
< 2.5 kg	−0.6 [−0.9, −0.3]***	−0.7 [−1.1, −0.4]***	−0.7 [−1.0, −0.3]***
Household-level factors			
*Maternal Factors*			
Mother's age			
25–34 (ref)	0	0	
15–24	−0.3 [−0.5, −0.1]**	−0.3 [−0.5, −0.1]*	
35 +	**−**0.1 [**−**0.3, 0.2]	**−**0.1 [**−**0.3, 0.2]	
Mother's education			
None (ref)		0	0
< Secondary		0.2 [**−**0.1, 0.5]	0.2 [**−**0.2, 0.5]
Secondary and tertiary		0.3 [**−**0.0, 0.6]	0.2 [**−**0.2, 0.6]
Mother co-residence			
Co-residing (ref)	0		
Not co-residing	0.6 [**−**0.1, 1.2]		
*Other household factors*			
Household head age			
35–49 (ref)		0	0
15–34		−0.3 [−0.6, −0.0]*	**−**0.3 [**−**0.7, **−**0.0]
50+ years		**−**0.1 [**−**0.3, 0.7]	**−**0.2 [**−**0.4, **−**0.0]
Community-level factors			
Area of residence			
> Predominantly South African (ref)	0	0	
> Predominantly Mozambican	**−**0.3 [**−**0.7, **−**0.0]	**−**0.3 [**−**0.7, 0.0]	

1 Only variables with significant association at 10% level of significance are shown.

* *p*<0.05

** *p*<0.01

*** *p*<0.001.

**Table 3:** Logistic regression analysis for predictors of stunting, underweight, and wasting among children aged 12–59 months (*n*=671), Agincourt, South Africa (2007) Variable^1^

	Stunting	Underweight	Wasted

	OR [95% CI]	OR [95% CI]	OR [95% CI]
Child-level factors			
HIV status			
Negative (ref)	1	1	1
Positive	2.3 [0.9, 5.6]	1.5 [0.5, 4.4]	1.9 [0.5, 6.5]
Unknown status	0.4 [0.1, 1.6]	0.3 [0.0, 2.2]	0.5 [0.07, 4.1]
Child age	0.6 [0.5, 0.7]***		
Birth weight			
≥ 2.5 kg (ref)	1	1	
< 2.5 kg	1.9 [0.9, 3.8]	3.1 [1.5, 6.4]**	
Household-level factors			
*Maternal factors*			
Mother's age			
25–34 (ref)	1		
15–24	1.6 [1.0, 2.6]		
35 +	1.1 [0.6, 1.9]		
Delivery place			
Health facility (ref)			1
Home			2.0 [1.0, 4.1]
Community-level factors			
Area of residence			
Predominantly South African (ref)	1	1	1
Predominantly Mozambican	2.2 [1.1, 4.3]*	2.0 [0.9, 4.4]	2.3 [0.9, 5.5]

1 Only variables with significant association at 10% level of significance are shown.

* *p* < 0.05

** *p* < 0.01

*** *p* < 0.001.

#### Caregiving experiences after learning a child's HIV status

These findings derive from in-depth interviews with caregivers of HIV-infected children 1 year after disclosure of the child's HIV status. Knowing the child's HIV status was perceived as beneficial and as enhancing the caregiver's competency in caregiving as it led to acquiring helpful advice from health professionals and other people. Reported changes included heightened hygiene, protecting other children from infection, heightened health-seeking behaviour, and improved child-feeding practices. Of importance was seeking ART for the HIV-infected children. Though most children were not on ART, knowing the child's HIV status stimulated the caregivers to seek ART for the child. Three children were on ART at the time of the follow-up study while eight other women had sought ART but the children had not been initiated due to medical reasons and other barriers, including financial, access, and social issues (21).

Caregivers of HIV-infected children faced barriers in caring for the children, including financial barriers, poor access to health services, and compromised physical ability to provide care due to their own poor health. Problems with health services included a shortage of drugs, for example, antibiotics for opportunistic infections, limited access to ARVs which were provided by only one health facility in the community distant from many of the villages, lack of confidentiality, and negative attitudes from health professionals, particularly at the nearby clinics. These barriers were reported to limit caregivers’ effective care (21).

## Discussion

### Patterns of malnutrition

We found a co-existence of substantial levels of undernutrition, particularly stunting at an early age, with marked levels of overweight/obesity and an elevated risk for metabolic disease in adolescent girls. The levels of early undernutrition in this rural community correspond with earlier findings in South Africa (18, 39, 40). This indicates persistence of the problem despite post-apartheid development. The higher prevalence of undernutrition amongst adolescent boys compared to girls is in line with findings in other South African studies (41). This differential prevalence of undernutrition by sex among adolescents is most likely due to a delay in the pubertal growth spurt in boys compared to the reference group, which occurs where undernutrition is prevalent (42). However, other factors may also contribute to these differences and may need further investigation. The finding of a higher prevalence of overweight/obesity and risk for metabolic disease among adolescent girls, and almost non-existent levels in adolescent boys, has been documented in other South African studies (19, 41), and in other LMICs (43). The prevalence of overweight/obesity in South African girls compares to that found in several upper-middle- and higher-income countries, whilst that of boys is generally lower (41).

### Factors associated with the double burden of malnutrition

As demonstrated in the conceptual framework (Fig. 1), the patterns of nutritional status observed may be influenced by nutrition transition and lifestyle changes, driven by changes such as economic growth, social change, and urbanisation occurring in South Africa (15, 16). These may work through other factors at individual, household, and community levels, some of which we have identified in this study (22, 23).

### Individual level factors

Undernutrition was associated with low birth-weight, HIV status, and child age. Low birth-weight, prevalent in the study population, may be a key reason why younger children were more likely to be stunted or underweight. While low birth-weight is an individual factor, it could also be interpreted as operating at household or community level as the health and nutritional status of the mother, which may be influenced by factors such as food security at the household or community level, plays a major role in determining birth-weight. HIV increases vulnerability to undernutrition directly for the infected child (44), or indirectly particularly due to decreased food security associated with lowered productivity or death of the bread winner (45). Consistent with other studies, obesity and risk for metabolic disease in the study participants were higher among girls, increased with increasing age and were positively associated with pubertal development (46). This may reflect the effect of factors such as increased sedentary behaviour and decreased physical activity with age and pubertal onset related to subsequent physical, social and emotional changes, particularly among girls (47). Several factors may explain the sex difference, including biological, behavioural, and social (48, 49). Biologically, energy needs differ for boys and girls and also in relation to rate of growth, and the timing of maturation differs by sex (48).

A link between early undernutrition and later obesity at the individual level in the study community may be explained by the developmental programming theory (50). Most of the adolescents, particularly those aged 15–20 years in our study setting, were born during or shortly after the apartheid era when nutritional deprivation due to political restrictions of the South African black population were apparent. Programming among children in deprived households may have occurred, while socio-economic changes and rapid urbanisation since 1994 may have increased access to food, particularly high dense foods, leading to obesity in later life. The difference in the prevalence of obesity between boys and girls needs further research.

### Household-level factors

At the household level, maternal age and age of household head were associated with young child undernutrition, while maternal age, household head's education level, food security, and SES were all associated with overweight/obesity and risk for metabolic disease among adolescents. While children of younger mothers and household heads were more likely to be undernourished, children of older mothers were more likely to be over nourished. We do not have enough data to explain these findings. However, we have a few postulations that need further research to guide interventions. Increased risk of undernutrition for children born of young mothers may relate to inexperience and inadequate childcare, or to biological characteristics (51). Age of the household head may be related to undernutrition through income and food security of the household, with younger household heads being potentially disadvantaged, due to having fewer resources. The higher risk of overnutrition among children of older mothers may be due to illiteracy (30), hence less knowledge of diet and the adverse health effects of obesity in the older age group, and possible stronger adherence to the cultural value ascribed to larger body size in women. Additionally, older mothers may monitor their child's behaviour less, which may affect the child's diet and physical activity patterns (26).

Education may affect nutritional status through knowledge of a proper diet and the harmful effects of overnutrition. Education level may also affect income levels, hence diet and sedentary lifestyle. In this study, a small amount of education was protective against overnutrition. On the other hand, the education level that would substantially affect SES in the study community (completed secondary and tertiary education) was not significantly associated, though it tended towards a positive association with obesity. The effect of education is mixed.

While we found a positive association between food security and overnutrition, available but limited literature on the association between food security and child/adolescent obesity is generally conflicting, and is often dependent on the level of development (52). Given a low food production base in the study area, food security may relate strongly to the ability to purchase food – in a society undergoing nutrition transition, this may generally mean energy-dense cheap processed foods. Likewise, the relationship between socio-economic status and childhood obesity varies across different populations depending on economic development (53). The positive relationship that we found is in keeping with some studies in LMICs, but conflicts with findings in many higher income countries (53). In this study setting, with high levels of poverty, SES may be related to affordability of purchased food items, including processed foods, while the poorer may generally rely on their limited farm products and wild foods. SES may also be related to overnutrition through sedentary behaviours related to, for example owning a car, hence walking less. Household members in higher SES households are likely to be involved in (circular) labour migration, commonly reported in the study area (28), which facilitates the transfer and introduction of urban practices to rural settings with consequent changes in diet (16).

### Community-level factors

The area of residence being a proxy for various factors, including environmental risks, availability of services, and shared cultures, emerged as a significant predictor of undernutrition. While the nationality of the mother per se was not associated with nutritional status, children living in villages mainly inhabited by people of Mozambican origin had poorer nutritional outcomes. These villages served as refugee settlements during and after the civil war in Mozambique from the early to mid-1980s. The villages have poor dwellings and infrastructure and are worse-off than predominantly South African villages with respect to basic services, including water, sanitation, electricity, and health facilities (54). Boys in the study community are generally more physically active compared to girls (data not shown) as indicated through a focus group discussion with community leaders (Group Discussion, Community Advisory Group, June 2008). This was also found in a national survey in South Africa in 2002 (19). As in many African countries, studies in South Africa have indicated that heavier bodies among females are preferred even during adolescence, particularly in rural settings (49), which may result in obesity among adolescent girls.

### Study limitations

We examined distal factors influencing a child's nutritional status at childhood, household, and community levels. We did not directly examine the proximal factors, including dietary intake, child's health status (apart from HIV status), physical activity patterns, and sedentary behaviour, which are also important in determining nutritional status, and need further investigation in future studies. The food security measurement tool was primarily designed as a simple tool to measure trends in household food security in the study area over time rather than to detail multiple dimensions of food security.

## Conclusion

This study has confirmed links between factors at different societal levels affecting nutritional status among children as portrayed in the conceptual framework presented (Fig. 1). The level of undernutrition that we have documented, particularly stunting at an early age in a country that has transitioned economically into a middle-income country, is worrying and may suggest the inadequacy or ineffectiveness of interventions that were put in place in the post-Apartheid era as a priority to address food insecurity and malnutrition. The substantial levels of overweight/obesity, particularly among adolescent girls, indicate that child growth and nutrition in rural South Africa is clearly shifting along the rural–urban continuum and is tending towards an urban-like profile. It is likely that this profile relates to changes in nutrition and dietary patterns in South Africa (15), as in other LMICs (1, 2). However, variation in other factors such as patterns of physical activity and social influences need to be investigated.

These findings have implications for public health policy and practice. The combination of early stunting and adolescent obesity may be an explosive combination associated with higher risk for obesity and cardio-metabolic diseases in adulthood (6). Childhood obesity and risk factors for metabolic diseases have been tracked into adulthood (6). The overweight/obesity prevalence in adolescents, particularly girls, may partly be contributing to the high levels of overweight/obesity reported in South African adults, particularly women (13). The substantial risk for metabolic disease in adolescent girls is of great public health importance as chronic diseases associated with obesity are already contributing markedly to the burden of disease in this community and other parts of South Africa among adults (10–12, 17).

These findings call for evidence-based interventions to alleviate the dual burden of malnutrition. With regard to obesity and metabolic disease risk, the study has identified predictors that may help in pointing out target groups for obesity prevention programs. Given the gender disparity in vulnerability to obesity (41), gender-sensitive programs targeted particularly to adolescent girls are needed. Although further research is required to clearly establish the proximate causes in this particular community, findings in other settings in South Africa (19, 46) suggest interventions should address physical inactivity, sedentary behaviour, and dietary patterns, particularly among adolescent girls. To address undernutrition, effective maternal interventions such as nutrition education and food programmes, are recommended. Interventions to improve nutritional outcomes of children infected or exposed to HIV may include targeted paediatric HIV screening and support to infected children. This study has demonstrated a high response rate and perceived usefulness of paediatric HIV testing with disclosure and counselling of caregivers on caregiving. However, several barriers highlighted in the results, including financial and health care access barriers, need to be addressed to reap the best from such an undertaking (21).
